# A Review of Carbon Anode Materials for Sodium-Ion Batteries: Key Materials, Sodium-Storage Mechanisms, Applications, and Large-Scale Design Principles

**DOI:** 10.3390/molecules29184331

**Published:** 2024-09-12

**Authors:** Qixing Jia, Zeyuan Li, Hulong Ruan, Dawei Luo, Junjun Wang, Zhiyu Ding, Lina Chen

**Affiliations:** 1 School of Materials Science and Engineering, Harbin Institute of Technology (Shenzhen), Shenzhen 518055, China; 2 Xinjiang Key Laboratory of High Value Green Utilization of Low-rank Coal, Changji 831100, China; 3 College of Physics and Materials Science, Changji University, Changji 831100, China; 4 School of Materials and Environmental Engineering, Shenzhen Polytechnic University, Shenzhen 518055, China

**Keywords:** carbon materials, sodium batteries, energy storage mechanism, large-scale application

## Abstract

Sodium-ion batteries (SIBs) have been proposed as a potential substitute for commercial lithium-ion batteries due to their excellent storage performance and cost-effectiveness. However, due to the substantial radius of sodium ions, there is an urgent need to develop anode materials with exemplary electrochemical characteristics, thereby enabling the fabrication of sodium-ion batteries with high energy density and rapid dynamics. Carbon materials are highly valued in the energy-storage field due to their diverse structures, low cost, and high reliability. This review comprehensively summarizes the typical structure; energy-storage mechanisms; and current development status of various carbon-based anode materials for SIBs, such as hard carbon, soft carbon, graphite, graphene, carbon nanotubes (CNTs), and porous carbon materials. This review also provides an overview of the current status and future development of related companies for sodium-ion batteries. Furthermore, it offers a summary and outlook on the challenges and opportunities associated with the design principles and large-scale production of carbon materials with high-energy-density requirements. This review offers an avenue for exploring outstanding improvement strategies for carbon materials, which can provide guidance for future application and research.

## 1. Introduction

As the energy demand continues to rise, the overexploitation of fossil fuels has resulted in environmental degradation and a struggle to meet modern energy needs. Consequently, the spotlight has shifted toward renewable energy sources, such as solar [1,2], hydroelectric [3], and biomass [4,5,6]. Nevertheless, their practical utilization is often hindered by geographical and environmental limitations, such as the common solar, wind, and hydroelectric energy need to rely on natural resources in a specific location to obtain a better energy collection [7,8]. Therefore, it is important to find an energy-storage system that is easy to prepare, convenient, and environmentally friendly.

Lithium-ion batteries (LIBs) are the most widely used form of energy storage due to their high energy density, long cycle life, and environmental friendliness. They have been extensively researched and applied in various aspects of daily life. However, there is a decreasing availability of lithium resources on Earth and the increasingly apparent limitations of LIB technology, particularly in effectively mitigating the problem of thermal runaway [9]. Lithium resources in nature are limited, and with the rapid development of new energy sources, the demand for lithium ore has led to extensive mining, causing a notable impact on the environment [10,11,12]. Furthermore, the rising cost of lithium ions has severely impacted the market development of LIBs [13]. Consequently, the pursuit of a new, sustainable energy-storage solution has become an imperative trend.

Sodium-ion batteries (SIBs), as an emerging energy storage technology, have garnered considerable attention owing to the physical and chemical properties resembling those of LIBs, along with their abundant availability on Earth and relatively lower cost [14,15]. Carbon materials, a pivotal component in SIBs, play a crucial role in determining battery performance [16,17,18]. This review aims to summarize the current research landscape of carbon materials for SIBs, briefly exploring the energy-storage mechanisms, comprehensively assessing the potential applications, and further analyzing the current challenges and opportunities for the industrialization of SIBs, as well as discussing the future development of the field.

## 2. Carbon Anode Materials for SIBs

**Figure 1:**
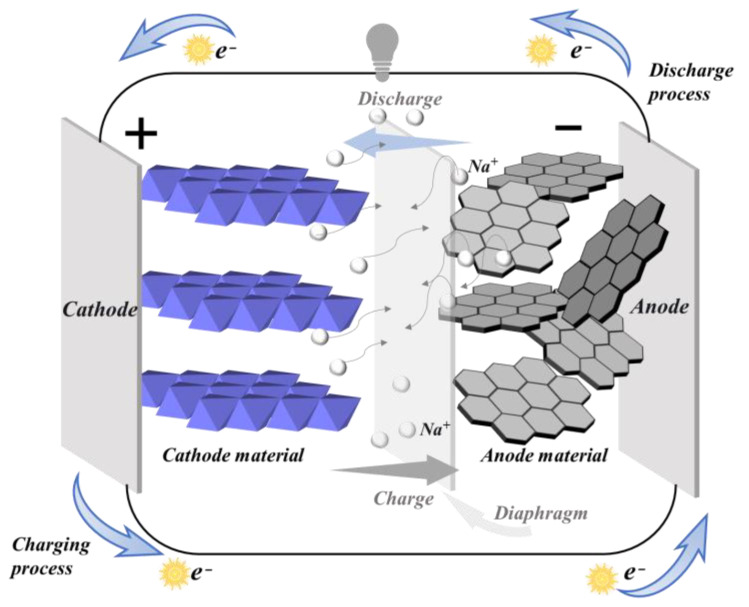
Diagram illustrating the operational principle of a SIB.

Sodium-ion batteries were initially proposed by Yao in 1967 [19], who discovered that Na^+^ could undergo rapid conduction in Na_2_O-11Al_2_O_3_. Armand and his team [20] presented the innovative concept of the “Rocking Chair Battery” at an Atlantic Treaty Organization meeting, significantly influencing subsequent research into SIBs, LIBs, and MIBs. The operational principles of SIBs are analogous to those of LIBs. The primary components of a sodium battery consist of an anode, a separator, an electrolyte, a cathode, and two current collectors. The separator’s function is to separate the positive and negative electrodes to avoid short circuits. The electrolyte permeates the separator to enable ion conduction, and the current collector is responsible for gathering and transporting electrons. As shown in Figure 1, during discharge, Na^+^ is emitted from the anode, penetrates through the separator, and intercalates into the cathode material, thereby enriching the cathode with sodium. During charging, the ion movement is reversed: Na^+^ ions are extracted from the cathode and intercalate back into the anode through the separator, resulting in the anode becoming sodium-rich and the cathode becoming sodium-poor. Throughout the charge and discharge cycles, corresponding redox reactions occur at the cathode and anode, respectively, allowing the movement of Na^+^ ions to maintain charge balance. The process is illustrated in the accompanying Figure 1. Employing Na_x_MO_2_ as the cathode and hard carbon (HC) as the anode, the reactions at both electrodes can be described by the equations below. Ideally, the insertion and extraction of Na^+^ ions leave the material structure unaffected, ensuring that the chemical reactions during charge and discharge processes are highly reversible.

Anode Reaction: $nC+y{Na}^{+}+ye^{-}⇌{Na}_{y}C_{n}$

Cathode Reaction: ${Na}_{x}{MO}_{2}⇌{Na}_{x-y}MO_{2}+y{Na}^{+}+ye^{-}$

Total reaction: ${Na}_{x}MO_{2}+nC⇌{Na}_{x-y}MO_{2}+{Na}_{y}C_{n}$

Given their abundance, cost-effectiveness, eco-friendliness, excellent electrical conductivity, and high capacity, carbon materials are pivotal as anode materials in SIBs [21]. The diversity of carbon materials is extensive, including hard carbon (HC) [22], soft carbon (SC) [23], graphite [24], graphene [25], carbon nanotubes (CNTs) [26], and porous carbon materials [27], among others [28,29]. Hard carbon and soft carbon are widely utilized in SIBs for their high energy density and superior cycling stability [28,30,31]. Graphene and CNTs have garnered considerable interest because of their large surface area and outstanding electrochemical properties [32,33]. Novel porous carbon materials, with their unique structures, offer abundant reactive sites and ion diffusion pathways, thus demonstrating enhanced sodium-storage capabilities and favorable cycling performance [34]. Consequently, investigating the use of carbon-based materials in SIBs is crucial.

### 2.1. Hard Carbon Material

#### 2.1.1. Definition and Characteristics

**Figure 2:**
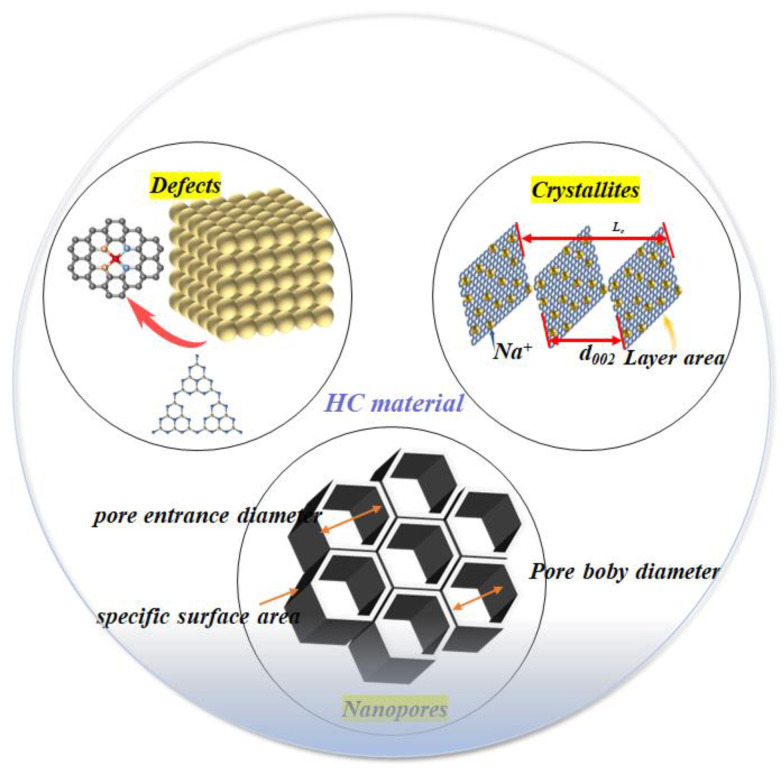
The main structure of HC.

Hard carbon is an amorphous carbon material that lacks long-range ordered crystal structures. The arrangement of carbon atoms in HC is disordered, and it exists in two forms: polycyclic aromatic structures and amorphous forms [35]. As shown in Figure 2, the production of hard carbon typically entails the pyrolysis of organic materials, including biomass and polymers, under conditions of oxygen-deficient or oxygen-free conditions [36]. Hard carbon was first named and modeled by Franklin in 1951, who proposed that non-graphitizable carbon possesses higher thermal and chemical stability compared to soft carbon, which can transform into graphite at high temperatures [37]. The characteristics of hard carbon include:

1. Nanopores: Hard carbon generally features nanopores at the nanoscale level, which enhance its specific surface area and offers additional active sites for sodium ion storage. The size and arrangement of these nanopores significantly impact the diffusion and storage efficiency of sodium ions [38]. Proper nanopore design can significantly improve the storage and release dynamics of sodium ions [30].
2. Defects: In hard carbon, defects like vacancies, edge dislocations, and grain boundaries serve as adsorption sites for sodium ions. These defects further enhance electron and ion mobility, leading to enhanced electrochemical performance in batteries [35].
3. Structural Attributes: Hard carbon’s distinctive structural features encompass a highly disordered carbon atom arrangement that provides numerous channels for sodium ion implantation and withdrawal, thus augmenting its Na-storage capacity. Moreover, the material’s layered architecture and expanded interlayer spacing promote swift migration and the efficient storage of sodium ions [39].

Hard carbon materials can be tailored for enhanced sodium-storage capabilities by manipulating synthesis conditions, such as the market for thermal treatment and the thermal decomposition temperature, which allows for the modulation of the size and distribution of the hard carbon particles [40,41,42]. Concurrently, doping and surface modification techniques can be employed to manage defect formation, thereby augmenting the number of sodium-storage sites. Moreover, tuning the interlayer spacing can lead to performance enhancements. The robust structural integrity of hard carbon, characterized by its high thermal and chemical stability, is pivotal when optimizing these materials. It is crucial to maintain the structural stability of the battery materials during the design phase. By implementing these structural optimizations, enhancing the sodium-storage capabilities of hard carbon materials can significantly expand their range of applications [43,44,45].

#### 2.1.2. Sodium-Storage Mechanism of HC

**Figure 3:**
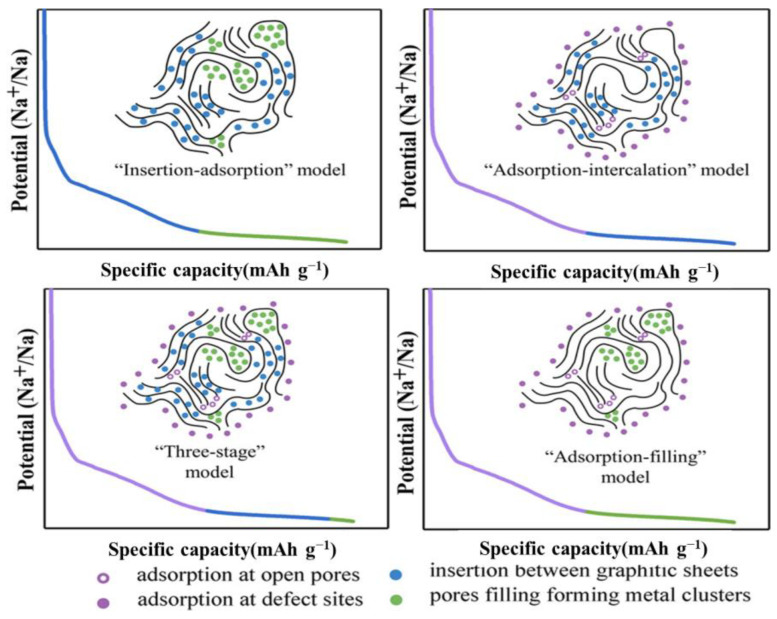
Four sodium-storage mechanisms of hard carbon. Reproduced with permission. Copyright 2022, Wiley [50].

Hard carbon is a form of carbon that remains non-graphitizable even at high temperatures. In contrast to graphite, hard carbon exhibits short-range order and contains partial graphitic domains within its structure. This internal structure leads to various characteristics, morphologies, and performance expressions in hard carbon materials prepared from different precursors [17,46,47,48]. The process of sodium ion storage in hard carbon is intricate and cannot be entirely described by a single model. Researchers have significantly advanced the comprehension of sodium storage in hard carbon materials, leading to notable progress in the field [28]. According to previous reports, researchers have divided the sodium storage process into three parts: capacitive adsorption, nanopore filling, and insertion into carbon interlayers [49]. As shown in Figure 3, four models have been proposed to explain this mechanism: the “Insertion-Adsorption” model [50], the “Adsorption-Intercalation” model [51], the “Three-Stage” model [52], and the “Adsorption-Filling” model [53]. The differences among these four sodium-storage mechanisms are shown in Table 1.

##### “Insertion-Adsorption” Model

D. A. Stevens and colleagues were pioneers in utilizing glucose for pyrolysis to synthesize HC and in elucidating its sodium-storage mechanism when used as an anode material for SIBs [37]. The way sodium is stored in hard carbon anodes for sodium-ion batteries resembles the lithium-ion insertion process in LIBs [54]. Specifically, sodium ion insertion and removal can be categorized into two distinct phases:

(1) The region with a gradual voltage change exhibits a hysteresis effect during the insertion and removal of sodium ions;
(2) The low potential plateau area is associated with the embedding of sodium or lithium ions into the nanopores between disordered layers, akin to an adsorption process [55].

**Figure 4:**
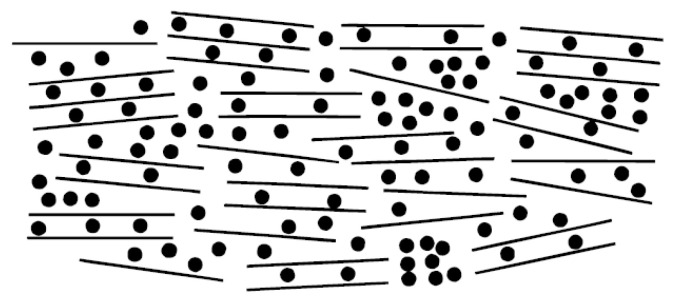
Layered structure model for Na-intercalated HC. Reproduced with permission. Copyright 2000, IOP Publishing [37].

The “house of cards” model mentioned in the report vividly describes the microstructure of hard carbon materials, as shown in Figure 4, where small aromatic units are arranged in a random, “house of cards”-like manner, forming nanopores. Na^+^ and Li^+^ insertion into hard carbon materials follows comparable processes involving intercalation between carbon layers and within nanopores.

##### “Adsorption-Intercalation” Model

Unfortunately, although the “Insertion-Adsorption” model offers an explanation for some sodium-storage behaviors in carbon materials, there are still discrepancies with this model in certain aspects. For instance, when the temperature of thermal decomposition is increased, the resulting carbon materials unexpectedly enhance sodium ion storage and broaden the low-voltage plateau segment [56], which does not conform to the explanation provided by the aforementioned model. The research results of Lu et al.’s [57] show that, following ball milling, hard carbon presents increased defects and reduced microcrystalline sizes, leading to a diminished capacity at low potentials and lower initial Coulombic efficiency (ICE). These findings further support the “Adsorption-Intercalation” mechanism. According to this mechanism, Na^+^ storage in hard carbon occurs in two stages [58]:

In the lower potential region, the reaction involves the corresponding intercalation and de-intercalation of Na^+^ between the graphite microcrystalline grains;

In the higher potential region, the reaction involves the transfer of charges on the surface of the microcrystalline clusters.

**Figure 5:**
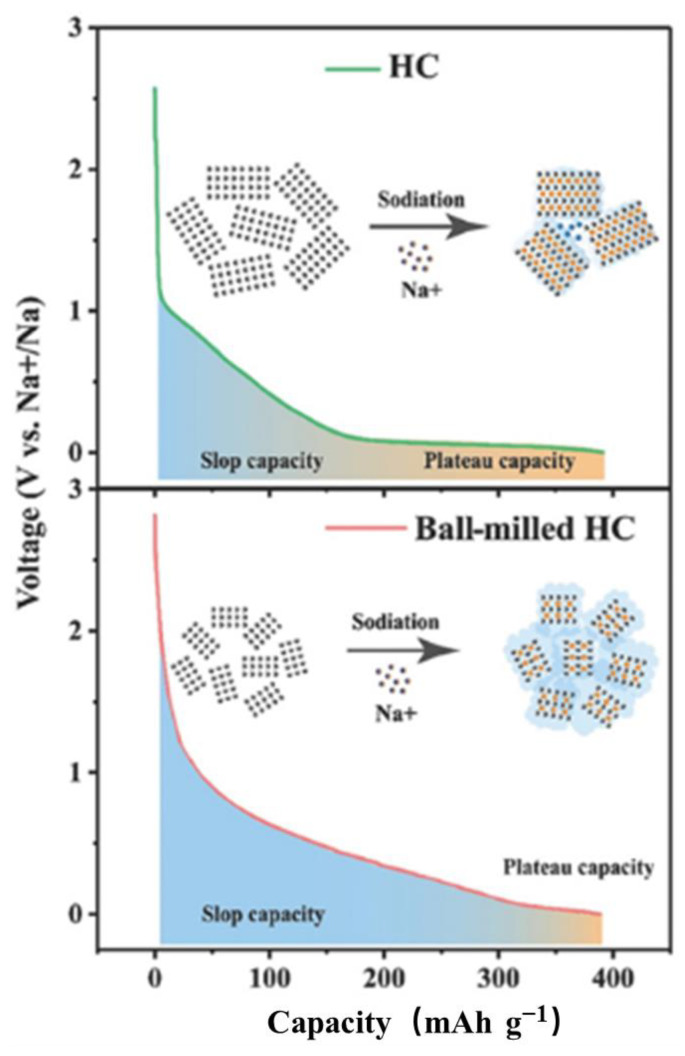
Ball milling and sodiation process of HC. Reproduced with permission. Copyright 2018, John Wiley and Sons [57].

This study also treated graphite electrodes using the ball-milling method and found that the longer the ball milling time, the lower the charging capacity of the graphite electrodes. As shown in Figure 5, this observation is consistent with phenomena observed in HC materials, further supporting the “Adsorption-Intercalation” mechanism.

##### “Three-Stage” Model

The two models described above, while capable of explaining a significant number of experimental phenomena, still leave some experimental observations unexplained. Based on the improvement in new characterization techniques and experimental protocols, researchers have gained new insights into the Na^+^-storage mechanism. Jin et al. [59] discovered a new “Three-Stage” structural model that has been proposed to explain the Na^+^-storage mechanism of a custom-designed HC material. They discovered a three-region sodium-ion storage behavior: chemical/physical adsorption (AC), intercalation between carbon layers (IC), and pore filling (FPC), which originate from the three phases of HC. In the AC phase, the active sites on the HC surface undergo adsorption of Na^+^, which includes edges, defects, and functional groups. In the IC stage, after the saturation of the surface adsorption during the AC stage described above, Na^+^ undergoes a new embedding mode into the graphite-like microchip layer, during which the diffusion coefficient (DC) of Na^+^ is changed. In the FPC stage, Na^+^ is embedded into the restricted space of graphite-like random stacks of HC, which further affects its diffusion in HC, and there is a rise in the DC of Na^+^ due to the high energy barrier of the restricted space.

**Figure 6:**
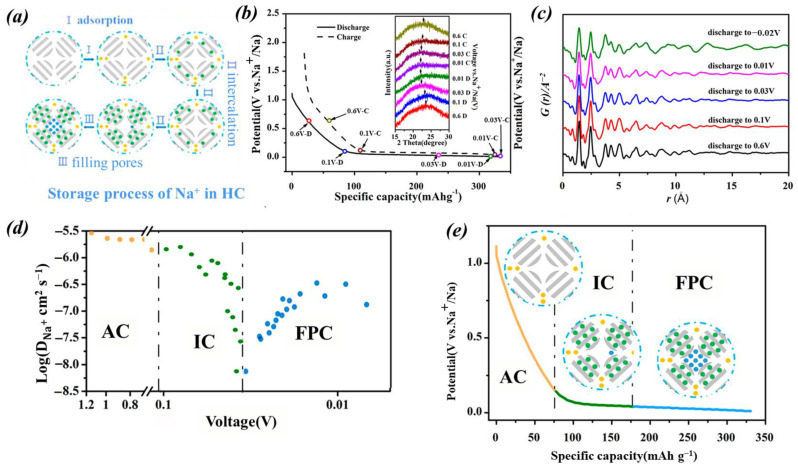
(**a**) Model diagram of sodium-storage mechanism in HC. Reproduced with permission. Copyright 2018, American Chemical Society; (**b**) EX XRD diagram during charge–discharge process. Reproduced with permission. Copyright 2018, American Chemical Society; (**c**) PDF diagrams during discharge at various stages. Reproduced with permission. Copyright 2018, American Chemical Society; (**d**) Na^+^ diffusion coefficient graph calculated by GITT. Reproduced with permission. Copyright 2018, American Chemical Society; (**e**) capacity schematic diagram for three stages of AC, IC, and FPC. Reproduced with permission. Copyright 2018, American Chemical Society [59].

Additionally, the reliability of this three-phase structural model was confirmed by the measurement of Na^+^ diffusion coefficient, XRD, and PDF (pair distribution function) analyses, as shown in Figure 6. This research also revealed the evolution of hard carbon structures under different carbonization conditions, thereby clarifying the relationship between their properties and structures and unveiling the sodium-storage mechanism.

##### “Adsorption Filling” Model

**Figure 7:**
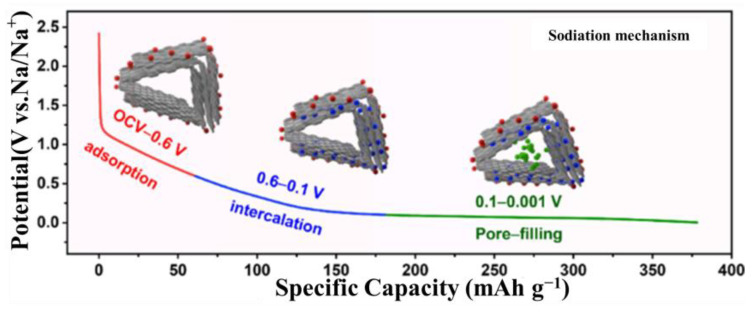
“Adsorption Filling” model for sodium-storage mechanism. Reproduced with permission. Copyright 2022, Elsevier [60].

Song et al. [60] discovered that by judiciously modulating the oxygen content, HC materials with diminished specific surface area and an increased fraction of pseudo-graphitic domains can be synthesized. This approach enables the attainment of a high sodium storage capacity and initial charge efficiency at a comparatively low pyrolysis temperature of 1100 °C. Concurrently, as shown in Figure 7, the Na^+^-storage behavior of these substances can be delineated into three distinct phases:

(1) The adsorption phase, where Na^+^ is held on the surface defects of the material during this stage (such as edges and vacancies);
(2) The insertion phase, where sodium ions insert into the pseudo-graphitic layers of the carbon material;
(3) The pore-filling phase, which occurs after the intercalation sites are saturated, and is characterized by the incorporation of Na^+^ into the closed pores [61].

#### 2.1.3. Application and Performance

**Figure 8:**
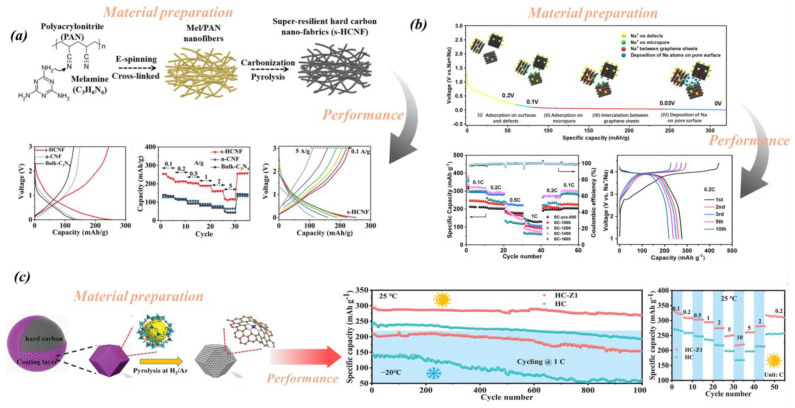
(**a**) Preparation and performance of S-HCNF. Reproduced with permission. Copyright 2020, John Wiley and Sons [62]. (**b**) Performance and sodium-storage behavior of SC-1400. Reproduced with permission. Copyright 2023, Springer Nature [47]. (**c**) Preparation process and performance of (HC-Z1). Reproduced with permission. Copyright 2024, John Wiley and Sons [63].

HC materials have unique chemical structures and electrochemical properties and offer numerous potential applications in SIBs. The amorphous form and large layer spacing of HC allow it to effectively embed and release Na^+^. For instance, Ding et al. [62] prepared HC nanofabrics with a helical nano-fiber structure via twisting and carbon treatment. The product of polyacrylonitrile (PAN) and polar substances such as melamine, as shown in Figure 8a, shows its preparation process and performance characteristics. With a current density of 2 A g^−1^, it exhibits a capacity of 1.05 mAh cm^−2^ and shows remarkable durability over multiple cycles. Wang et al. [47] conducted research by pyrolyzing sawdust at various temperatures to prepare a series of HC. As shown in Figure 8b, the HC derived from sawdust processed at 1400 °C (SC-1400) exhibits optimized electrochemical characteristics, with an initial reversible electrode capacity as high as 309.2 mAh g^−1^ (at 0.1 C, equivalent to 30 mA g^−1^). In the beginning, the material exhibits CE of 83.4%; after 100 charge–discharge cycles, its capacity decreases by 4.2% compared to the original. Song et al. [63] created an HC material (HC-Z1) by incorporating CoN_4_ sites onto the surface of commercially available HC particles. As shown in Figure 8c, this innovative material demonstrates good performance, reaching a specific capacity of 220.6 mAh g^−1^ at 25 °C. Furthermore, it exhibits a remarkable reversible capacity of 288.7 mAh g^−1^ and maintains a storage capacity of 270 mAh g^−1^, even at the extremely cold temperature of −40 °C. In this study, the synthesis of bent CoN_4_ structures was found to enhance charge transfer kinetics by facilitating the decomposition of NaPF_6_ in the diglyme electrolyte, resulting in the formation of a thin and uniform NaF solid electrolyte interface (SEI). The development of a dense, NaF-rich SEI significantly reduced both the ion transport resistance (R_sei_) and the charge transfer resistance (R_ct_). The localized electric field effectively promoted the adsorption of Na^+^ ions onto the carbon surface, thereby accelerating the otherwise sluggish desolvation process and enhancing the diffusion of Na^+^ ions at low temperatures. Generally speaking, the desolvation and migration capabilities of Na^+^ ions at the electrolyte/electrode interface are pivotal in determining the kinetic rate of charge transfer at high current densities and low temperatures. Consequently, strategies aimed at further enhancing the desolvation and migration abilities of Na^+^ ions warrant further investigation.

Different preparation methods lead to variations in the synthesized hard carbon materials. Due to the shrinkage that occurs during the pyrolysis process, the resulting hard carbon has a higher density. Differences in density affect the performance of hard carbon as an anode material for sodium-ion batteries [64]. HC with higher density may exhibit better electrochemical performance, but the shrinkage phenomenon could lead to greater resistance in mechanical strength and ion transport. Hard carbon materials obtained through solution processing have more controllable microstructures, especially affecting ion transport by influencing the size, shape, and uniformity of the pore distribution. In contrast, materials obtained by pyrolyzing sawdust have a relatively rough surface, and irregular pore structures may further limit their performance. Considering environmental impacts, the solution processing method may produce chemical waste during the process, increasing preparation costs and potentially hindering recycling. On the other hand, the method of pyrolyzing sawdust produces process products with better environmental sustainability.

The amorphous state and large layer spacing of hard carbon materials enable effective Na^+^ embedding and release, making them a better choice for anode materials. The ideal negative electrode material can be better designed via different preparation means, such as adding the introduction of active sites or preparing synthetic materials. As researchers continue to explore the mechanism and optimize the preparation method, the subsequent HC will be even better in sodium storage.

### 2.2. Soft Carbon Materials

#### 2.2.1. Definition and Characteristics of SC

The structure of soft carbon materials contrasts with that of hard carbon and is generally transformed from amorphous or pseudo-graphitic carbon structures into graphite structures through heat treatment at temperatures below 1000 °C [31]. During the thermal processing, the microstructure of soft carbon evolves from amorphous or pseudo-graphitic configurations into a more ordered graphite structure, concurrently experiencing volume contraction and an increase in density. The production of soft carbon materials can be sourced from natural origins, such as coal tar and petroleum coke, or from artificial sources, including polymers and biomass. These materials are fabricated using techniques such as pyrolysis [65], CVD [66], and the sol-gel process [67].

#### 2.2.2. Sodium Intercalation Mechanism

**Figure 9:**
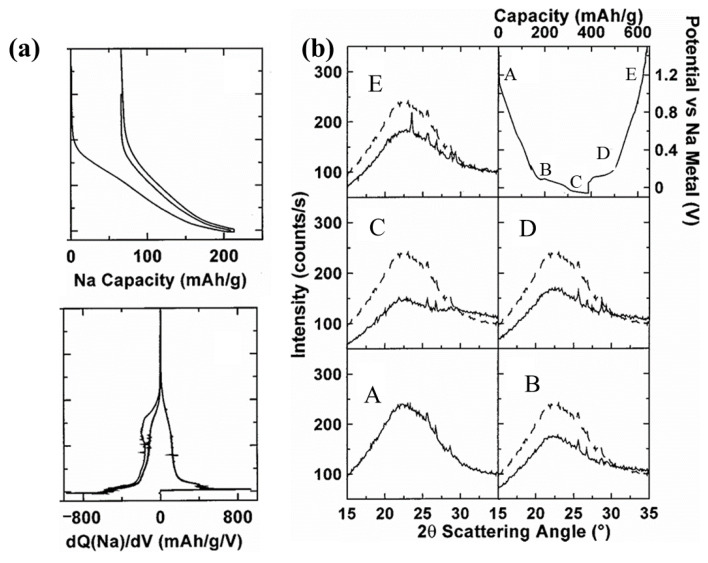
(**a**) Sodium intercalation behavior, (**b**) in situ X-ray scattering studies of Na intercalation into SC, the dashed parts in figures (A–E) represent the first scan, and the top right corner shows the approximate locations of the points. Reproduced with permission. Copyright 1948, IOP Publishing [68].

Stevens et al. [68] observed that when Li and Na are embedded into carbon materials, the interlayer spacing between adjacent layers expands. As shown in Figure 9b, broad-angle in situ XRD investigations have corroborated the potential of Li and Na to penetrate the interlayer gaps of these disordered carbon materials. Furthermore, small-angle in situ scattering studies have distinctly demonstrated the embedding of Li and Na into the nanopores of disordered HC. Jian et al. [69] observed that the addition of Na^+^ into soft carbon materials results in an irreversible expansion of the interlayer spacing, which is particularly pronounced during the initial sodium-ion intercalation phase. Na^+^ intercalation into soft carbon results in an increase in interlayer spacing from 3.6 Å to 4.2 Å., and this expanded spacing remains irreversible even after the subsequent desodiation process.

As indicated in Figure 9a, the storage mechanism of Na^+^ is intricately linked to the local defect structure of SC. The presence of defects in SC leads to high binding energy for Na^+^ during the intercalation process, which may account for the emergence of an irreversible quasi-plateau during the initial intercalation phase. Cheng et al. [31] determined that carbon materials with adjustable microstructures can be synthesized by varying the proportions of PER and TPC in the Friedel–Crafts reaction. The storage mechanism of Na^+^ in soft carbon materials adheres to the “intercalation/defect adsorption-closed pore filling” model. In the inclined section of the charge–discharge curve, Na^+^ is stored via intercalation reactions and defect adsorption, whereas in the plateau region, they fill the closed pores. Owing to its narrower interlayer spacing and fewer closed pores, soft carbon materials predominantly store Na^+^ through intercalation reactions and defect adsorption. Conversely, hard carbon materials, characterized by their wider interlayer spacing and a greater abundance of closed pores, are capable of attaining a higher Na^+^-storage capacity.

In summation, a cohesive model that universally explains the Na-storage approach in SC materials has yet to be formulated. Furthermore, the mechanisms become increasingly intricate to interpret when intricate modifications and doping processes are applied to carbon materials. Consequently, the need for sophisticated characterization technologies and computational approaches for optimization becomes imperative.

#### 2.2.3. Application of Soft Carbon in SIBs

**Figure 10:**
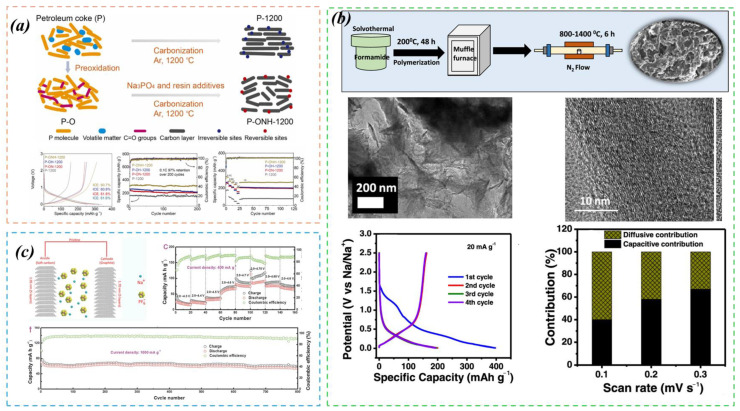
(**a**) Preparation process and performance characteristics of p-1200. Reproduced with permission. Copyright 2024, Elsevier [23]. (**b**) The synthesis process of NC, along with its SEM image, charge–discharge performance graph, and pseudocapacitive contribution rate graph. Reproduced with permission. Copyright 2022, American Chemical Society [73]. (**c**) The model diagram of NDIBs and the rate and cycling performance of soft carbon as the negative electrode. Reproduced with permission. Copyright 2017, John Wiley and Sons [72].

In SIBs, the lamellar structure of SC materials permits the insertion and deintercalation of Na^+^ within the layers, thus facilitating the storage and liberation of electrical energy [70]. The kinetics of this insertion and deintercalation process significantly influence the battery’s charge–discharge characteristics. The lamellar architecture of soft carbon materials contributes to an elevated migration rate of Na^+^, which in turn improves the battery’s rate capability and cycle stability [65,67,71]. Wu et al. [23] successfully developed a SC anode material that markedly enhances the ICE and carbon yield. The binder facilitates an enhancement in micropores and inhibits graphitization, while sodium phosphate assists in expanding the interlayer spacing and enhancing reversible groups. This, in turn, fosters the crosslinking of graphite microcrystals and introduces additional sodium, thereby augmenting ICE and conductivity. As illustrated in Figure 10a, the synergistic effects of these additives culminate in the optimized sample demonstrating an exceptional reversible storage capacity of 311.9 mAh g^−1^ in SIBs, coupled with excellent cycle life, an ICE of 90.7%, and a carbon yield of 70%. Fan et al. pioneered the use of SC as the anode and graphite as the negative electrode in sodium-based dual-ion batteries, marking the first instance of such an application. As shown in Figure 10c, these batteries exhibit a discharge voltage of 3.58 V, coupled with outstanding discharge performance at 103 mAh g^−1^, exceptional rate characteristics, and remarkable durable cycling performance, maintaining 81.8% of their capacity over 800 cycles [72]. Mishra et al. [73] successfully produced nitrogen-doped soft carbons by tailoring the nitrogen content and graphitic character via annealing temperatures extending from 800 °C to 1400 °C. As shown in Figure 10b, these materials obtain a significant specific capacity of approximately 201 mAh g^−1^ at 20 mA g^−1^. Moreover, they exhibit outstanding cycling stability, maintaining 87% of their initial reversible capacity following 500 cycles at 100 mA g^−1^.

SC materials obtained through different preparation methods exhibit varying performances. The synthesis of SC through the thermal polymerization of pyromellitic dianhydride (PTCDA) compound is characterized by its good scalability, making it suitable for large-scale production. By controlling the thermal polymerization conditions during the synthesis process, the microstructure of the SC can be optimized, thereby influencing its performance in SIBs. The synthesis of nitrogen-doped SC from formamide via a solvothermal process followed by high-temperature annealing is straightforward and allows for the adjustment of nitrogen doping and graphitization, which facilitates the potential optimization of the material’s performance; this method is also more environmentally friendly.

In SIBs, the lamellar structure of SC materials enables efficient sodium ion insertion and extraction, thereby enhancing overall battery performance. Advanced SC anodic electrode materials notably boost initial CE and carbon yield, showcasing exceptional charge capacity and cycling stability. SC holds substantial promise for sodium-ion battery applications, and further enhancements in energy density and cycling stability can be realized by refining the structure of carbon materials, such as adjusting the levels of nitrogen or other dopants and graphitization. Investigating novel synthesis techniques and surface modifications of carbon materials could also potentially elevate the performance of soft carbon in SIBs, broadening its application scope. Compared to hard carbon materials, soft carbon materials have a more stable structure. Soft carbon materials possess a higher degree of graphitization, with a more regular internal arrangement, and their interlayer spacing is relatively larger, which facilitates the intercalation/deintercalation of ions. In contrast, hard carbon materials have an amorphous structure and a lower degree of graphitization, with a relatively disordered arrangement of carbon atoms. From the perspective of cycling stability, soft carbon, due to its better structure, maintains its structure more effectively during the intercalation/deintercalation of Na^+^. Unfortunately, compared to the preparation cost and environmental friendliness of hard carbon materials, the production of soft carbon is still complex and can be destructive to the environment. Therefore, developing new methods for synthesizing soft carbon is crucial, providing new ideas for future researchers to improve upon.

### 2.3. Graphite and Graphene Materials

#### 2.3.1. Structure and Properties of Graphite and Graphene

**Figure 11:**
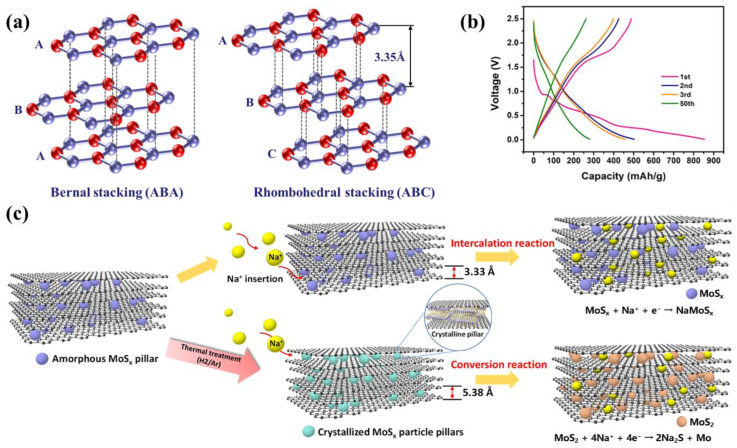
(**a**) Schematic diagram of graphite layered structure. Reproduced with permission. Copyright 2021, American Chemical Society; (**b**) charge/discharge voltage curve of EG-MoSx-900; Reproduced with permission. Copyright 2021, American Chemical Society; (**c**) reaction mechanism diagram for sodium storage of EG-MoS_x_. Reproduced with permission. Copyright 2021, American Chemical Society [78].

The structure of graphite is made of carbon atoms in a hexagonal mesh plane regular parallel stacking of layer structure crystals. Within the graphite crystal, carbon atoms within the same layer form covalent bonds with adjacent carbon atoms through sp^2^ hybridized orbitals, with each carbon atom being connected to three other atoms via these covalent bonds. Graphite exhibits two types of stacking configurations: Bernal (ABA) stacking and Rhombohedral (ABC) stacking (as illustrated in Figure 11a). Graphite is often referred to as a potential anode material for sodium-ion batteries because of its success in high-performance lithium-ion battery applications due to its energy density (with the theoretical capacity of 372 mAh g^−1^) and low cost [74]. However, the ionic radius of lithium ions is 0.76 Å, whereas that of sodium ions is 1.06 Å [75], larger than lithium ions. This larger radius results in only a limited amount of Na ions being accommodated within the interlayer spacing of graphite (3.35 Å), translating into a reversible capacity that falls below the standard performance (35 mAh g^−1^) compared to the theoretical capacity for lithium-ion intercalation in graphite [76]. This suggests that graphite cannot serve as an optimal anode material for sodium-ion batteries in its conventional form [77]. To address this issue, researchers have made progress in increasing the interlayer spacing to facilitate sodium-ion insertion. For instance, Kim et al. [78] tackled the problem of irreversible capacity loss in graphite for sodium-ion battery applications by creating expanded graphite (EG). They synthesized a graphite material (EG-MoSx) incorporating MoSx pillars, with a storage mechanism depicted in Figure 11c. This approach yielded a graphite anode material featuring an expanded interlayer spacing of 5.38 Å. When used with ether-based electrolytes and after calcination at 900 °C, the anode exhibited a second discharge capacity of 501 mAh g⁻^1^ (as shown in Figure 11b), along with high cycling stability. This strategy could also provide insights into expanding interlayer spacing for other alkaline-ion batteries.

**Figure 12:**
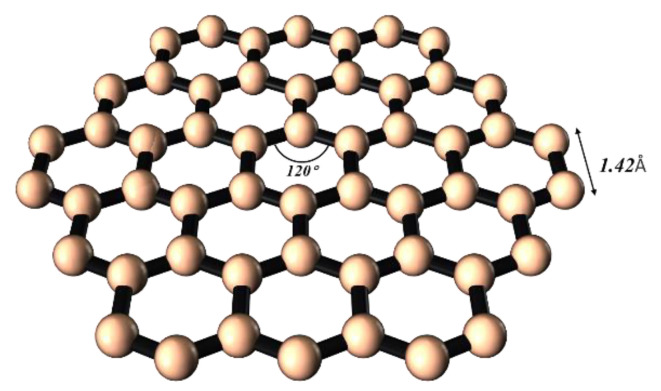
Graphene structure diagram.

As shown in Figure 12, graphene, once a hot material of a cross-era [79], is structured with a single layer of carbon atoms arranged into a honeycomb hexagonal lattice, connected by σ bonds formed through sp^2^ hybridization, and its structure is maintained by van der Waals forces. Graphene possesses good electrical conductivity, mechanical strength, and thermal conductivity, vastly broadening its application horizons in realms such as electronic devices [80], composite materials [81], and energy-storage solutions [82].

Hard carbon and soft carbon are amorphous carbon materials, where HC stands out for its microcrystalline and amorphous microstructure domains, endowed with electrochemical activity and robust electrochemical stability, rendering it an optimal candidate for the anode electrodes of LIBs and SIBs. With its impeccable layered structure and remarkable physical and chemical attributes, graphene holds significant promise across a multitude of fields. Conversely, hard carbon and soft carbon, distinguished by their cost-effectiveness and steadfast electrochemical performance, are pivotal in the domain of electrochemical energy storage. Despite graphene’s planar structure offering a relatively high surface area and the presence of defects that serve as additional sites for Na^+^ adsorption, its application in SIBs has been limited due to factors such as preparation costs and structural intricacies. The sodium-storage capacity of graphene ranges from 150 to 350 mAh g^−1^; as an anode material for sodium-ion batteries, the initial Coulombic efficiency (ICE) of unmodified graphene is between 60 and 80%. After modification, the ICE of graphene materials (82.3%) [83] is also lower than that of hard carbon materials (92%) [84]. Due to the limited mention of ICE in the literature for soft carbon materials in the application of sodium-ion batteries, their ICE values are typically similar to those of hard carbon materials. The reaction potential of graphene in sodium-ion batteries (0.1 V–0.3 V) [85,86] is greater than that of soft carbon/hard carbon (0.05 V–0.2 V) [31,87], and the reaction potential directly affects the energy of sodium ions during the reaction process, thereby impacting performance. The synthesis of graphene is more complex and difficult compared to that of soft carbon/hard carbon, with stringent control conditions required during its process. The application of graphene in sodium-ion batteries still faces significant challenges.

#### 2.3.2. Sodium-Storage Mechanism of Graphite and Graphene

**Figure 13:**
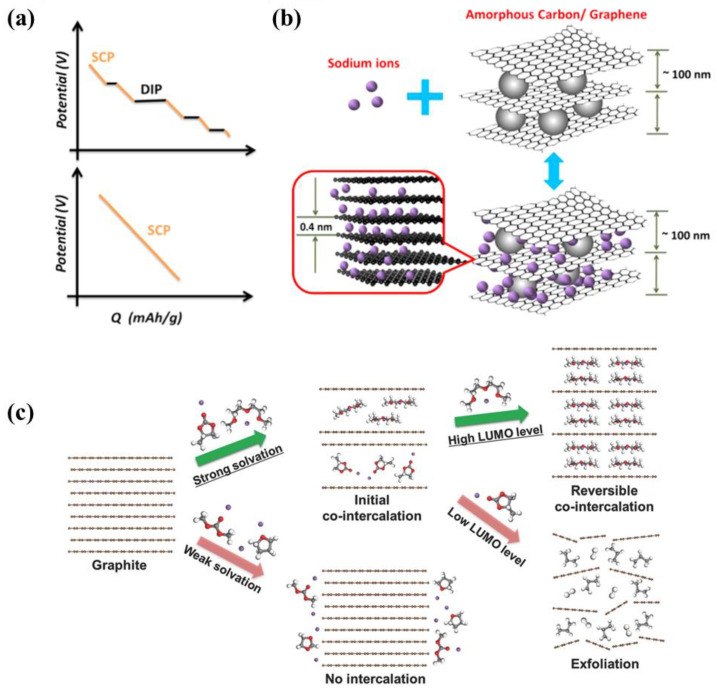
(**a**) Discharge curves of DIP/SCP hybrid electrode and SCP electrode. Reproduced with permission. Copyright 2015 Elsevier; (**b**) schematic diagram of AC/G sodium storage mechanism. Reproduced with permission. Copyright 2015, Elsevier [88]. (**c**) Schematic illustration of the conditions for sodium-solvent co-insertion in graphite. Reproduced with permission. Copyright 2016, John Wiley and Sons [93].

The structural variations among different carbon materials exert a substantial influence on their performance characteristics. Nonetheless, the majority of carbon materials share a comparable sodium-storage mechanism, which is predicated on an intercalation-adsorption paradigm. As shown in Figure 13a,b, the research literature has identified that the Na^+^-storage mechanism of graphene encompasses two principal components: the diffusion-controlled intercalation process (DIP) and the surface-induced capacitive process (SCP) [88]. Specifically, the DIP involves Na^+^ diffusing into the interlayer spaces of graphene [89]. Given the inherent spacing between graphene layers, Na^+^ is able to intercalate into these interlayer regions. This insertion typically transpires within a lower voltage regime and is contingent upon the diffusion rate of Na^+^ within graphene layers. The DIP process significantly contributes to the capacity of SIBs. However, its slower kinetics impose limitations on the battery’s performance at high rates. Conversely, the SCP entails the adsorption and capacitive effects of Na^+^ on the surface of graphene. In the SCP process, sodium ions are not only intercalated between the graphene layers but also adsorbed onto defects, nanopores, and functional groups situated on the surface of graphene [90]. This adsorption process, akin to the charge–discharge dynamics of a capacitor, exhibits rapid kinetics, enabling swift charge and discharge capabilities.

To enable sodium storage in graphite, several specialized treatments are required [91], akin to the previously mentioned expansion of graphite. This expansion, resulting from an enlarged interlayer spacing, facilitates the efficient storage of sodium ions through intercalation reactions. Lee et al. [92] reduced the graphite particle size via ball milling, producing ball-milled graphite with an expanded interlayer spacing of 0.36 nm. This material exhibited remarkable Na-storage performance, delivering a reversible capacity of 290.5 mAh g^−1^ at a current density of 0.01 A g^−1^ and maintaining stability over 3000 cycles. Beyond merely increasing the interlayer spacing, the selection of an appropriate electrolyte can also facilitate the insertion of sodium ions into graphite layers through co-intercalation with solvent molecules (Figure 13c) [93]. With the assistance of specific solvents, sodium ions can be reversibly stored within graphite [94,95,96].

#### 2.3.3. Application of Graphene and Graphite

**Figure 14:**
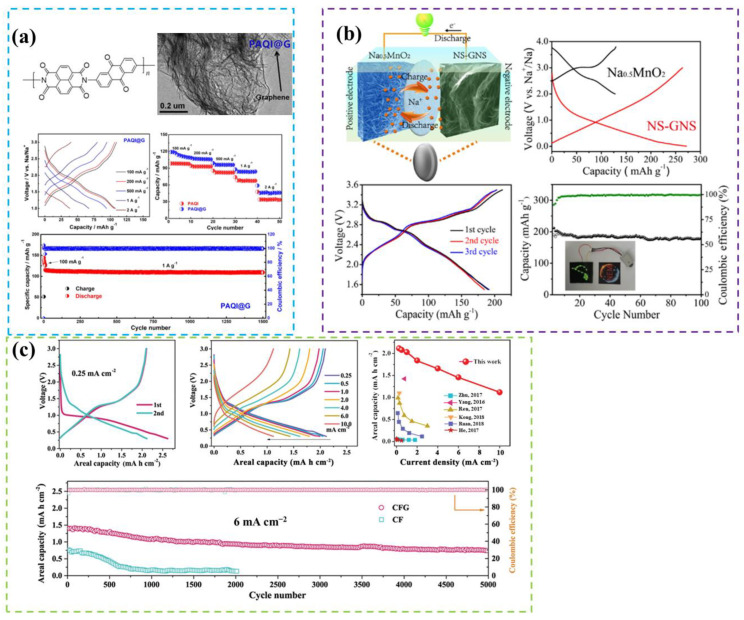
(**a**) Structure and performance of PAQI@G. Reproduced with permission. Copyright 2020, Elsevier [101]. (**b**) Performance illustration of NS-GNS. Reproduced with permission. Copyright 2018 Elsevier [100]. (**c**) Electrochemical performance of sodium-ion battery with flexible freestanding graphene electrode. Reproduced with permission. Copyright 2019 John Wiley and Sons [102,104,105,106,107,108,109].

Graphene, a groundbreaking 2D material, is held together exclusively by weak van der Waals interactions between its layers, endowing graphene with exceptional conductivity, mechanical strength [97], and thermal conductivity. These properties greatly expand the potential applications of graphene in fields such as electronic devices, composite materials, and energy storage. The application of graphite in energy storage is mainly in LIBs, and in SIBs, its Na-storage capacity is low. Asher et al. [98] succeeded in fully reacting graphite and sodium via the gas-phase method, but less Na was found to enter into the graphite layer in the resultant product. The graphite layer gap is small compared to its other materials, the radius of Na^+^ is larger than its gap, and more energy is required in the process of embedding so that reversible embedding/relocation cannot take place within the effective voltage window. Theoretical calculations show that the reason for the lower sodium-storage capacity of graphite is due to thermodynamic factors and its inability to form stable compounds with Na^+^. Therefore, in SIBs, graphite has a limited use scenario in its anode material and is not widely used [99]. Based on previous work, Figure 14a,b demonstrate the enhanced performance achieved through ion doping [100,101]. Graphene, with its impeccable layered structure and outstanding physical and chemical properties, exhibits significant application prospects across various fields. However, when used as an anode for sodium-ion batteries, it still poses challenges, such as low initial Coulombic efficiency and poor cycling stability. Chen et al. [102] designed and synthesized a three-dimensional hierarchical graphene anode network through surface modification and heat treatment. This electrode boasts ample electron and ion-transport channels, exceptional structural stability, and favorable interfacial properties, effectively achieving a high areal capacity of 2.12 mAh cm^−2^ at 0.25 mA cm^−2^ with an impressive initial Coulombic efficiency of 81%. Furthermore, it exhibits high-rate anode performance, delivering 1.12 mAh cm^−2^ at 10 mA cm^−2^, and remarkable cycling stability, retaining 95% of its capacity after 500 cycles (Figure 14c). Exploring better intercalation materials to further enhance the performance of sodium-ion batteries represents a future trend. Graphite, constrained by its layered structure, exhibits inferior performance compared to its analog in lithium-ion batteries [103]. Nevertheless, integrating various improvement strategies, such as expanding interlayer spacing and selecting more suitable electrolytes to facilitate the co-intercalation of sodium ions, could enhance the amount of sodium ion intercalation within graphite, thereby addressing its issue of low reversible capacity. Meanwhile, HC and SC, owing to their cost-effectiveness and stable electrochemical characteristics, play a pivotal role in the realm of electrochemical energy storage.

### 2.4. Other Carbon-Based Materials

**Figure 15:**
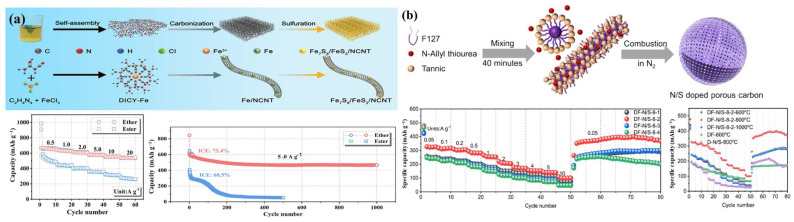
(**a**) Preparation and performance of the Fe_7_S_8_/FeS_2_ heterostructure. Reproduced with permission. Copyright 2023, Springer [114]. (**b**) Preparation process and performance of the DF-N/S material. Reproduced with permission. Copyright 2024, Elsevier [112].

Apart from the previously mentioned HC, SC, and graphene materials, CNTs, porous materials, and various modified carbon materials are also employed as anode materials for SIBs [26,110,111]. Zhang et al. successfully synthesized porous carbon spheres as anode materials for SIBs using a soft template-induced self-assembly approach, resulting in the creation of N and S dual-doped porous carbon spheres (DF-N/S) [112]. As shown in Figure 15b, at 0.05 A g^−1^, the DF-N/S material exhibits a remarkable specific capacity of 327.04 mAh g^−1^. The Na-storage mechanism of the DF-N/S material is characterized by a diffusion-controlled insertion process and a surface-induced capacitive process [113]. Sodium ions permeate into the interstices of the porous carbon spheres and are adsorbed onto surface defects, nanoholes, and functional groups. This adsorption process, akin to the charge–discharge dynamics of a capacitor, offers swift kinetics, enabling rapid charge and discharge capabilities. The porous architecture and N/S dual-doping attributes of the DF-N/S material enhance the storage capacity for Na^+^ and the electrochemical performance of the battery, conferring upon it a high Na^+^ storage and commendable cycling stability within ether-based electrolytes.

Song et al. crafted a heterostructure composed of iron sulfides utilizing nitrogen-doped CNTs (Fe_7_S_8_/FeS_2_/NCNT) via an in situ pyrolysis and sulfidation technique [114]. This material electrode displays exceptional Na-storage capabilities in ester-based electrolytes, as shown in Figure 15a, characterized by a high reversible capacity of 403.2 mAh g^−1^ over 100 cycles, impressive rate performance with 273.4 mAh g^−1^ at 20.0 A g^−1^, and robust long-term cycling stability, maintaining 466.7 mAh g^−1^ at 5.0 A g^−1^ after 1000 cycles [115]. The Na-storage mechanism of the Fe_7_S_8_/FeS_2_/NCNT electrode is predominantly ascribed to its distinctive heterostructure, which furnishes abundant lattice defects and ample buffer space, thereby enhancing the electrode’s electronic conductivity and structural robustness [116].

## 3. The Current Status and Development of SIBs Industrialization

**Figure 16:**
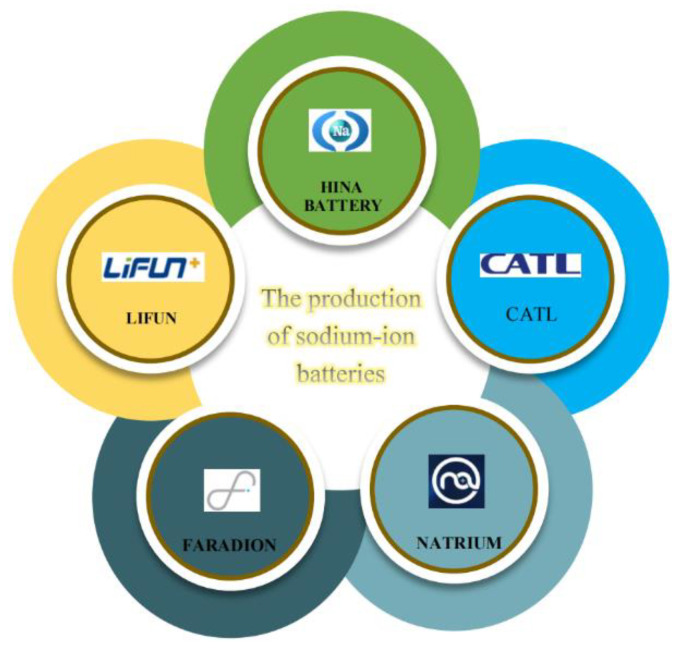
The industrialization companies of SIBs.

Currently, the industrialization companies of SIBs include HiNa Battery Technology Co., Ltd. (HiNa BATTERY) in Beijing, China; Contemporary Amperex Technology Co., Ltd. (CATL) in Ningde, China; Zhejiang Sodium Innovation Energy Co., Ltd. (NATRIUM) in Shaoxing, China; Li-Fun Technology Co., Ltd. (LIFUN) in Zhuzhou, China; and FARADION in Sheffield, United Kingdom (Figure 16). All of these companies employ the use of carbon materials in the production of anode and cathode components for SIBs. Among these, the products of the HiNa BATTERY employ a copper-based oxide layer as the anode and a coal-based amorphous carbon (soft carbon) as the cathode. These products exhibit several advantageous characteristics, including longevity, a wide temperature range, high power, and mass production. The sodium-ion battery developed by FARADION is a layered metal oxide/hard carbon organic electrolyte system that also employs hard carbon as the anode material [117]. The anode material for SIBs developed by CATL also exhibits a distinctive pore structure and is composed of hard carbon. Furthermore, the anode materials for SIBs produced by NATRIUM and LIFUN are also composed of hard carbon. Hence, it can be inferred that carbon materials are more suitable for use as anode materials for SIBs, which also indicates that significant progress is likely to be made in the development of carbon materials for SIBs.

Given the similarities between the fundamental principles of lithium and sodium batteries, the former can inform the latter’s industrialization trajectory. Furthermore, the cost of SIBs is comparatively advantageous when compared to LIBs. The anode materials used in SIBs are typically derived from low-cost and abundant sources, including sodium, iron, manganese, copper, and other elements. The anode material represents a significant portion of the cost of sodium batteries, accounting for approximately 16%. Various anode materials are employed in SIBs, including metal compounds, carbonaceous materials, alloy compositions, and non-metallic monomers. Among these, carbon-based materials have surfaced as the most promising candidates for anode materials in SIBs, offering a diverse range of sources, robust sodium-storage capacity, and competitive cost. Nevertheless, there are evident shortcomings, including the low first Coulombic efficiency, cycle life, and structural consistency.

To address the aforementioned challenges associated with hard carbon materials, a range of strategies can be employed, including pre-sodiumation, structural modulation, interface structure, and the selection of suitable precursor materials as a carbon source. Nonetheless, the transition from theory to practice necessitates the consolidation of technological advancements. Consequently, the technology associated with SIBs is still in its infancy and is yet to overcome the current technical challenges. Furthermore, it is anticipated that the sodium-ion battery market will continue to expand as the technology matures and costs decline. From an application perspective, in addition to electric automobiles and energy-storage systems, SIBs have the potential to be utilized in a variety of fields, including smart grids, communication base stations, drones, and others. The aforementioned fields have disparate requirements pertaining to battery performance and cost. Conversely, the adaptability and customizability of SIBs will facilitate their extensive market penetration in these fields.

## 4. Design Principles of Carbon Materials for Large Scale-Up

### 4.1. Design Principles

Carbon materials, renowned for their unique physicochemical properties such as porosity, exceptional conductivity, stability, and tunable pore architectures, demonstrate significant potential in the applications of energy-storage devices. Despite the current involvement of numerous companies in the production of SIBs, researchers have intensified their endeavors over the past few years to enhance the electrochemical attributes of carbon anode materials. This pursuit aims to facilitate the upscaling of carbon materials for utilization in commercial and diverse other applications [118].

#### 4.1.1. Electrochemical Performance Optimization

In SIBs, the optimization of the electrochemical properties of carbon materials, as one of the key electrode materials, holds paramount importance for bolstering the comprehensive performance of the batteries [119]. Researchers have adopted various modification methodologies, encompassing pore structure modulation, surface modification, heteroatom doping, structural innovation, etc. [120].

**Figure 17:**
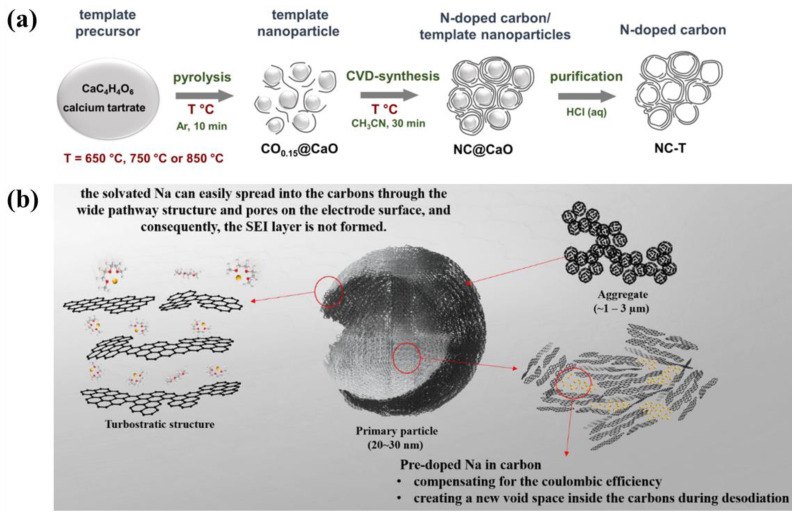
(**a**) Synthesis schematic of n-doped carbon materials at different temperatures. Reproduced with permission. Copyright 2023, MDPI [121]. (**b**) Schematics showing concept of Na-C structure. Reproduced with permission. Copyright 2024, Elsevier [122].

Fedoseeva et al. [121] modulated the nitrogen speciation, porosity, and specific surface area of the carbon material through the manipulation of synthesis temperature (Figure 17a), enhancing its performance and enabling good energy storage in sodium-ion and lithium-ion battery applications. Kang et al. [122] enhanced the Coulombic efficiency of SIBs during discharge by doping sodium during carbon synthesis (Figure 17b), concurrently creating voids to facilitate efficient sodium ion insertion/extraction, thereby boosting Coulombic efficiency and reversible capacity during cycling.

Shao et al. [123] improved the performance of electrochemical capacitors by enhancing the carbon pore structure. This improvement in the pore structure enhanced the electrochemical capacitor performance. Designing carbon architectures characterized by distinct morphology and structure, including nanosheets, nanofibers, and three-dimensional porous structures, can shorten the ionic diffusion routes and enhance the material’s surface-to-volume ratio as well as its utilization efficiency.

#### 4.1.2. Cost–Benefit Analysis of Carbon Materials

**Figure 18:**
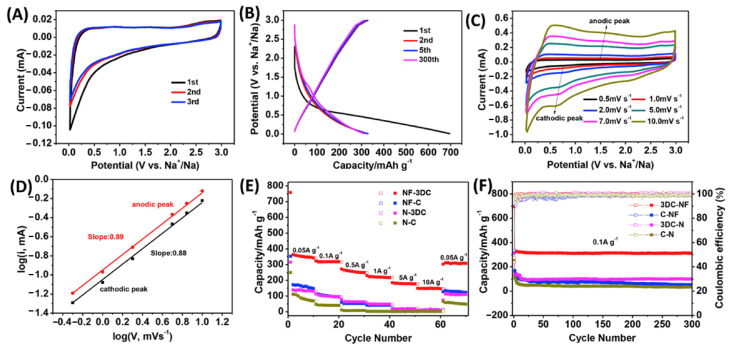
Electrochemical performance of NF-3DC as a SIB anode. (**A**) CV curves of NF-3DC at 0.1 mV s^−1^; (**B**) Charge/discharge curves of NF-3DC in SIB for Na metal at 0.1 A g^−1^; (**C**) CV curves of NF-3DC at different scan rates; (**D**) log i vs log ν plots for oxidation/reduction states; (**E**) rate performance between 0.05 and 10 A g^−1^; (**F**) cycling performance of NF-3DC, NF-C, N-3DC and N-C at 0.1 A g^−1^. Reproduced with permission. Copyright 2020, Elsevier [124].

Advanced carbon materials like CNTs and graphene exhibit superior properties yet are initially cost-intensive due to complex synthesis and material constraints. However, technological advancements and economies of scale have mitigated costs. Notably, laboratory-synthesized electrode materials offer cost-effectiveness and enhanced performance over commercially acquired counterparts, underscoring a favorable cost–benefit analysis for carbon materials. He et al. synthesized a novel F and N co-doped 3D carbon self-assembled sheet (NF-3DC) by using polytetrafluoroethylene, N-containing resins, and KOH, which was used as a cathode material for a sodium-ion battery, at a 346.45 mAh g^−1^ capacity at a current density of 0.05 A/g [124,125]. Figure 18 shows that the SIBs produced by this convenient and low-cost method still have good electrochemical performance. Sharma et al. synthesized Z@Cdots-ZnO/Carbon nanocomposites as acceptors for solar cells prepared utilizing a straightforward microwave-facilitated hydrothermal synthesis approach, which showed a small difference in performance when compared to cells prepared from devices using fullerenes, but the material cost exceeded 50-fold, significantly impacting the cost-effectiveness of the Z@Cdots devices. The cost efficiency or the economic viability of Z@Cdots devices is significantly superior to that of devices utilizing fullerenes as acceptors [126].

In evaluating the cost-effectiveness of carbonaceous substances for storing energy, it is essential to consider the total cost, encompassing material cost, processing cost, and battery capacity efficiency [28,127,128]. A comprehensive comparison of the overall costs associated with different materials can provide substantial support for the optimal design of energy-storage systems. It is anticipated that as technology progresses and large-scale production becomes a reality, the cost of carbon materials will decline further, thereby facilitating their deployment and advancement across a broader spectrum of applications. Concurrently, further research is imperative into refining the preparation procedures and optimizing the performance strategies of carbon materials, with the aim of augmenting their overall advantages in the realm of energy storage.

#### 4.1.3. Environmental and Sustainability Considerations

**Figure 19:**
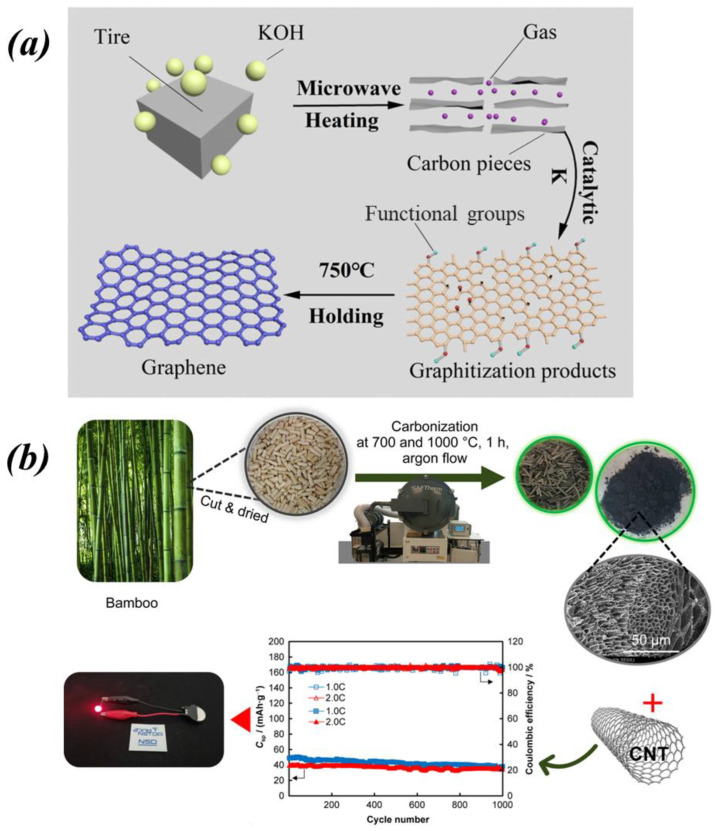
(**a**) Schematic illustration of waste tires into graphite. Reproduced with permission. Copyright 2022 Springer Nature [133]. (**b**) Schematic illustration of bamboo converted into carbon materials for sodium-ion battery. Reproduced with permission. Copyright 2022 Elsevier [134].

In the pursuit of a sustainable energy future, the utilization of environmentally friendly, resourceful, and renewable materials is of paramount importance. SIBs have emerged as the leading contenders for energy storage, demonstrating considerable promise in both energy storage and conversion capabilities in the current era [129]. They have the potential to significantly alleviate the shortage of resources in conventional LIBs and have a broader development prospect [130]. Hard carbon possesses a distinctive advantage as a pivotal material for SIB anodes, characterized by a vast array of accessible raw material sources and a commendable sustainability profile. It is noteworthy that the preparation of hard carbon can effectively utilize biological wastes as raw materials. In the past, such materials were often regarded as useless burdens, casually discarded, or incinerated. This practice not only resulted in the waste of valuable resources but also contributed to environmental pollution [131]. However, through the application of advanced conversion technologies, these biowastes, which were previously regarded as useless, can be transformed into valuable resources for carbon material production. Diverse categories of biowastes, including crop residues, forestry waste, food processing waste, and old tires, can be collected and converted into carbon electrodes for batteries [132]. Wu et al. [133] conducted thermogravimetric analysis and dielectric property tests on waste tires and their mixtures and observed that when the temperature surpassed 750 °C, potassium ions catalyzed the reduction reaction between the soft carbon present in the tires and the carbonaceous material, yielding potassium vapor as a byproduct. Subsequently, this potassium vapor acted as a catalyst to promote the formation of porous graphite and graphene from the carbon in the tires (the synthesis process is depicted in Figure 19a). Pothaya et al. [134] converted bamboo into hard carbon using a high-temperature treatment followed by a low-temperature process and prepared a stable sodium-ion battery anode by compositing it with carbon nanotubes. This anode exhibited 78% capacity retention after 1000 cycles at 1C (Figure 19b). The synthesis of carbon from a variety of inexpensive and readily available sources renders it an economically viable material, offering opportunities for customizing its properties [46].

**Figure 20:**
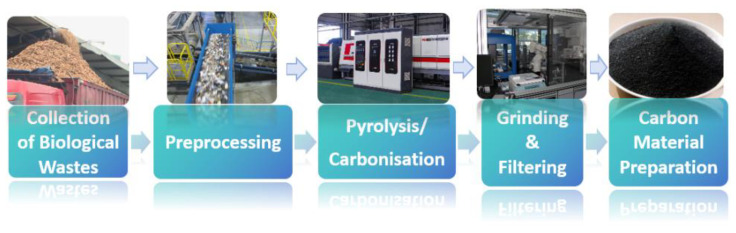
Bio-waste to carbon material flowchart.

Waste can be converted into usable carbon materials by following a process (Figure 20). Preprocessing is the initial treatment of collected biowaste, such as the removal of impurities, crushing, drying, etc., for subsequent processing. Pyrolysis/carbonization is the pyrolysis or carbonization of pre-treated biowaste under a controlled temperature and atmosphere to convert it into carbon-rich materials [135]. Subsequently, the carbonized material is ground to the desired particle size, and non-conforming particles are removed via filtering. Finally, the material is processed into carbon material of the desired composition.

This transformation has the dual benefit of realizing the resourceful utilization of waste and mitigating environmental pollution. Furthermore, it embodies the principles of the circular economy [136]. By converting bio-waste into high-performance energy-storage materials, we can promote the green transformation of energy technology and contribute to environmental protection and sustainable development. Consequently, the utilization of bio-waste to prepare carbon materials is a promising and potential path worthy of further research and exploration.

### 4.2. Challenges of Scaling Applications

In response to the escalating worldwide appetite for environmentally sustainable energy sources, SIBs have emerged as a pivotal research area in energy storage, owing to their potential advantages over LIBs: abundant resource availability and lower production costs. In this wave of development, carbon materials have been transformed into various structures and integrated with other ions to enhance the electrochemical efficiency of SIBs, such as porous carbon nanofiber structures [137] and different atoms co-doped porous carbon structures [130], wherein the properties of the carbon material are crucial for augmenting the overall energy efficiency of the battery system.

Despite the immense application potential of carbon materials across multiple fields, transitioning them from laboratory research to large-scale commercialization faces numerous challenges. These challenges encompass not only the optimization of the material’s inherent properties but also encompass issues related to production costs, environmental impact, and technical integration [138,139,140]. The preparation processes of carbon materials are often intricate, particularly when striving for high-purity, high-performance materials. Although research and development efforts by scientists in recent years have somewhat reduced the manufacturing costs of carbon materials, cost remains a significant barrier to their competitiveness in large-scale applications [141,142]. Consequently, the development of low-cost, high-efficiency production processes remains crucial for expanding the application scale of carbon materials. Successfully applying carbon materials in specific domains necessitates effective integration with other technologies, encompassing compatibility with existing production equipment, compositing with other materials, and customized development tailored to specific application requirements [143]. The complexity of technological integration and the diversity of application developments add to the difficulties in scaling up the application of carbon materials. Concurrently, adhering to the principle of environmental protection throughout the production process is imperative, aiming to minimize the environmental impact of both the production and use of carbon materials. This involves recycling waste materials and achieving green production and utilization.

**Figure 21:**
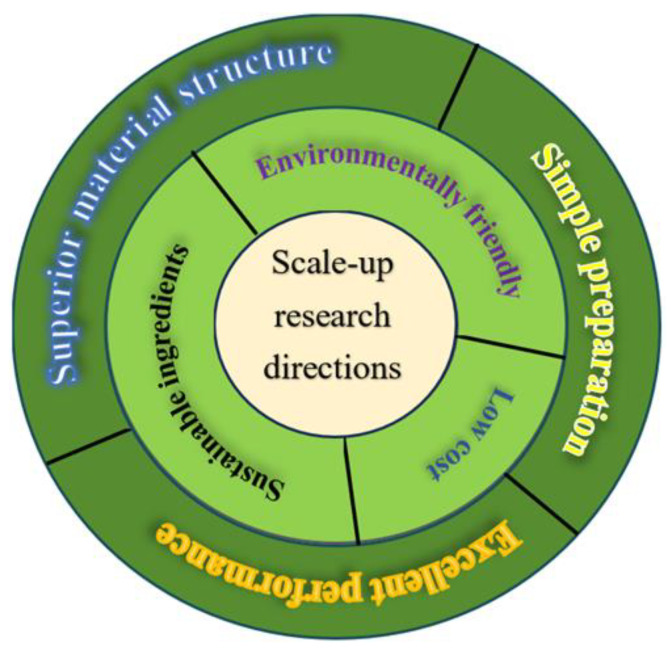
Scale-up research directions.

The mass production technology of carbon materials serves as the foundation for realizing commercial applications, and porous carbon materials have previously been prepared using a complex multi-step template method, which constrains large-scale production and thus prompts the pursuit of simple and economical methods for the preparation of porous carbon materials [144]. The large radius of sodium ions inevitably imposes higher requirements on the electrode materials employed in SIBs [145]. Figure 21 illustrates the research development directions for scale-up applications, emphasizing the need to optimize production costs and efficiency via technological advancements and process refinement, which constitutes a pivotal development path for scaling up carbon material production technology.

The carbon material, being a pivotal constituent of the anode, is crucial to its performance. In parallel, the cathode material, serving as the other essential end of the battery for storing and releasing sodium ions, possesses chemical properties, crystal structure, and ion diffusion rates that directly dictate the energy density, cycling longevity, and safety characteristics of the battery [146].

## 5. Conclusions and Prospects

As the world continues to develop, traditional fossil energy sources are increasingly unable to satisfy people’s energy demands, and the environmental damage caused by energy extraction is becoming increasingly serious [147,148,149]. In recent years, the development of renewable energy sources has been rapid, with the development of SIBs standing out as a notable achievement. Consequently, numerous companies have embarked on the production of electrode components specifically for SIBs.

Despite the potential of carbon materials in sodium-ion batteries, there are still many limitations to their application that could affect their large-scale production. The following section provides a summary.

1. ICE

Compared to the near 100% Coulombic efficiency of aqueous zinc-ion batteries, the ICE (Initial Coulombic Efficiency) of carbon materials in sodium-ion batteries is somewhat lower. The reasons for this may include the irreversible sodium ion intercalation/deintercalation process during the first charge–discharge cycle, which generates an SEI (Solid Electrolyte Interface layer) on the cathode side. This process also reduces the effective concentration of Na+ and leads to electrolyte loss. During the intercalation process, carbon materials can expand in structure, resulting in damage to the electrode structure. Although this can create new active sites, the irreversible capacity loss is significant. Researchers are continuously innovating to improve ICE: optimizing the pretreatment and surface modification of carbon materials, such as through heat treatment, to reduce surface active sites and inhibit the occurrence of irreversible reactions, as well as adding electrolyte additives like fluorides and sulfides to promote the formation of a more stable SEI layer. Material innovation, such as using 3D printing to fabricate cathode materials, can regulate structural voids to make the Na^+^ intercalation/deintercalation process smoother. Composite electrode materials are also a way to address low ICE by doping with heteroatoms (P, S, etc.) or using nanostructured carbon to enhance conductivity and mechanical properties.

2. Rate/Cycle Performance

Compared to lithium-ion batteries, the rate performance of sodium-ion batteries remains a significant barrier to their further large-scale production and application. The larger radius of sodium ions makes their intercalation process more difficult, especially at high rates. The SEI layer has a significant impact on the cycling stability of sodium-ion batteries. The larger ion radius leads to more complex reactions with the electrolyte, resulting in an SEI layer that may thicken or crack during cycling. The disruption of this SEI layer directly affects the durability of the cycling process. The structure of carbon materials themselves also affects their cycling performance. During cycling, carbon materials undergo structural changes, such as graphitization or transitioning from ordered to disordered structures. Conductivity is another factor; unmodified carbon materials have poor conductivity, which leads to higher internal resistance during high-rate charge–discharge processes, limiting performance. To address these issues, the following approaches can be used: composite carbon materials, doping carbon materials, or synthesizing new carbon materials with more stable structures, which can lead to the formation of a denser SEI film during ion transport. Another solution is to develop new electrolyte compositions that improve contact with electrode materials and reduce the occurrence of side reactions.

3. SEI

The SEI layer has a significant impact on the cycling and rate performance of sodium-ion batteries. In sodium-ion batteries, the formation of the SEI layer is not a simple superposition of reactions; due to the larger diameter of sodium ions affecting kinetic behavior, the reaction process is less controllable, making the formation of the SEI more complex. The composition of the electrolyte also influences the formation of the SEI layer; if the electrochemical window of the electrolyte is unstable, the resulting SEI layer may not have a stable structure. The SEI layer is typically composed of various inorganic and organic compounds, and the physical and chemical properties of this complex layer affect the stability of the structure, making it more unstable compared to lithium-ion batteries. Sodium ions have a slower diffusion rate, leading to an uneven formation of the SEI layer, which can cause the stress concentration during cycling, challenging the structure. Improving the SEI layer will help address the performance issues of carbon-based materials in sodium-ion batteries.

The utilization of carbon materials as anodes in SIBs demonstrates significant potential and offers broad prospects for the future. Different types of carbon materials exhibit distinct characteristics. The microstructure of hard carbon renders it electrochemically active and exhibits strong electrochemical stability, making it a prime candidate for anodes in both lithium-ion and SIBs. In contrast, soft carbon possesses a more ordered structure than hard carbon and possesses the unique capability to transform into graphite at elevated temperatures. The electrochemical properties of soft carbon can be tailored through the precise adjustment of heat treatment parameters [150], enabling it to display tailored properties for specific applications. Both hard and soft carbons are pivotal in the realm of electrochemical energy storage owing to their robust electrochemical properties and favorable cost-effectiveness. Graphene, with its impeccable layered structure and exceptional physical and chemical properties, holds immense promise across multiple domains. However, its adoption in SIBs is constrained by factors such as fabrication costs and structural intricacies. By enhancing the performance of carbon materials via structural optimization strategies (including pore structure modulation, surface modification, and ion doping) [56], the rapid intercalation/deintercalation of sodium ions can be facilitated, thereby enhancing their energy-storage capabilities in SIBs.

Through continuous technological innovation and optimization, carbon materials are anticipated to achieve large-scale application in the realm of SIBs, thereby facilitating the commercialization and sustainable development of these batteries and making significant contributions to the advancement of energy-storage technology [151,152,153,154]. It is foreseeable that the design of carbon materials will become increasingly refined and intelligent with the swift development of novel technologies. Additionally, the development and utilization of composite materials offer novel avenues for the implementation of carbon materials in SIBs [155]. As SIB technology continues to mature and costs further decrease, its industrialization process will progressively deepen. In the foreseeable future, carbon materials will exhibit broader application prospects in the field of SIBs, contributing to the widespread adoption and sustainable development of renewable energy.

## Figures and Tables

**Table 1:** Comparison of four sodium-storage mechanisms in HC.

Model Name	Core Concept	Main Processes	Features	Distinctions
Insertion–Adsorption Model	Adsorption and intercalation of sodium ions on the surface and in nanopores of hard carbon	Sodium ion adsorption Nanopore intercalation	Describes the adsorption and intercalation of sodium ions on the surface and within nanopores of hard carbon	Emphasizes the adsorption role of sodium ions on the surface and within nanopores
Adsorption–Intercalation Model	Adsorption and intercalation of sodium ions within defects and microcrystalline sizes of hard carbon	Intercalation between layers Charge transfer on microcrystalline clusters	Describes the adsorption and intercalation of sodium ions within defects and microcrystalline sizes of hard carbon	Highlights the impact of defects and microcrystalline sizes on sodium ion storage
Three-Stage Model	Chemical/physical adsorption, intercalation between layers, and pore filling of sodium ions in hard carbon	Chemical/physical adsorption Intercalation between layers Pore filling	Proposes three distinct stages of sodium ion storage	Clearly distinguishes between chemical/physical adsorption, interlayer intercalation, and pore filling
Adsorption Filling Model	Optimizing hard carbon material structure by adjusting oxygen content to enhance sodium ion storage performance	Sodium ion adsorption Intercalation between layers Pore filling	Describes the adsorption, intercalation, and pore filling of sodium ions in hard carbon materials	Focuses on optimizing hard carbon material structure through oxygen content adjustment to improve sodium ion storage performance
